# Supplementary material for: Crinamine Induces Apoptosis and Inhibits Proliferation, Migration, and Angiogenesis in Cervical Cancer SiHa Cells
Source: Biomolecules. 2019 Sep 16;9(9):494. doi: 10.3390/biom9090494 (PMC6770758; doi:10.3390/biom9090494)
Supplement: Supplementary file 1 [file biomolecules-09-00494-s001.pdf]

Table 1. Primers used for RT-qPCR.

Primer name	Sequence	T_a (°C)
SNAI1	Bio-Rad assay ID: qHsaCED0057267	60
TWIST1	Bio-Rad assay ID: qHsaCED0043959	60
ZEB1	Bio-Rad assay ID: qHsaCED0045418	60
ZEB2	Bio-Rad assay ID: qHsaCED0038149	60
VIM	Bio-Rad assay ID: qHsaCED0042034	60
MMP1	Bio-Rad assay ID: qHsaCID0017039	60
RPS13	Bio-Rad assay ID: qHsaCID0038672	60
U6-F	5′-CTCGCTTCGGCAGCACATATAC-3′	62.9
U6-R	5′-GGAACGCTTCACGAATTTGC-3′	62.9
AURKA	Bio-Rad assay ID: qHsaCID0022123	60
AURKB	Bio-Rad assay ID: qHsaCID0005962	60
AKT1	Bio-Rad assay ID: qHsaCID0011338	60
BCL2-F	5′-CGACTTCGCCGAGATGTCC-3′	60
BCL2-R	5′-CACACATGACCCCACCGAAC-3′	60
BCL2L1-F	5′-CACTGTGCGTGGAAAGCGT-3′	60
BCL2L1-R	5′-CTCTAGGTGGTCATTCAGGTAAGTG-3′	60
BIRC5	Bio-Rad assay ID: qHsaCED0001615	60
BUB1B	Bio-Rad assay ID: qHsaCID0022007	60
CCNA2	Bio-Rad assay ID: qHsaCID0017452	60
CCNB1	Bio-Rad assay ID: qHsaCED0044529	60
CCND1-F	5′-CCTCGGTGTCCTACTTCAAATGTG-3′	62.9
CCND1-R	5′-GTTCCTCGCAGACCTCCAGC-3′	62.9
CDC20	Bio-Rad assay ID: qHsaCID0012637	60
CDK4-F	5′-GGACATATCTGGACAAGGCACC-3′	62.9
CDK4-R	5′-ACTGTTCCACCACTTGTCACCAG-3′	62.9
EGFR	Bio-Rad assay ID: qHsaCID0007564	60
MELK	Bio-Rad assay ID: qHsaCID0010246	60
PBK	Bio-Rad assay ID: qHsaCID0014389	60
PLK1	Bio-Rad assay ID: qHsaCED0046411	60
PTTG1	Bio-Rad assay ID: qHsaCID0038120	60
RHOA	Bio-Rad assay ID: qHsaCED0042259	60
TOP2A	Bio-Rad assay ID: qHsaCID0006279	60
VEGFA	Bio-Rad assay ID: qHsaCED0043454	60
PGK1	Bio-Rad assay ID: qHsaCED0042912	60
